# Ninth International Medical Congress

**Published:** 1887-09

**Authors:** 


### EDITORIAL.

### NINTH INTERNATIONAL MEDICAL CONGRESS.

The issue of the present number of the *Journal and Examiner* has been delayed beyond the usual time for its appearance because of our desire to present as full a report of the transactions of the International Medical Congress held in Washington, as might be found to be practicable in so short a time. This we have been enabled to do through the enterprise of the press, medical and secular. Much of the report here given has been obtained through the courtesy of the well-known publishing house of Messrs. William Wood & Company, of New York, from advanced proofs of their publication, the *Medical Record.*

As is now well known, the attendance at the congress was very large, nearly three thousand names having been registered.

The work of the general sessions was of a high order of merit, and there was a noteworthy recognition of the dignity and the importance of the occasion, so that the sessions were characterized by decorum which was befitting the greatness of the occasion. A brief summary of the general addresses delivered is given in our report.

In the sections, the work done manifested in a marked manner the interest which the medical profession of all countries had taken in this congress. Many, who from various causes had been prevented from being personally in attendance, sent their contributions of valuable papers, and some of the sections had more papers presented to them than could be read and discussed in the time allotted them, although there were two sessions a day in many of the sections. In consequence of this, some papers were read in other sections than those for which they were originally intended. As so often happens in the large assemblings of medical men, valuable papers are only finished on the eve of the convening of the meeting, and are presented at too late a period to be read during the sessions. Such proved to be the case during this congress, and some of them will only appear in the report of the transactions, and abstracts of them can not now be obtained.

Although the congress convened in the capital at an unfavorable season of the year, socially, yet the Committee of Arrangements made commendable effort to have the occasion as pleasant as possible for the visitors from other countries, during their stay in Washington, and, after the adjournment of the congress, special excursions were provided for their enjoyment.

